# Trust, Care Avoidance, and Care Experiences among Kenyan Women Who Delivered during the COVID-19 Pandemic

**DOI:** 10.1080/23288604.2022.2156043

**Published:** 2022-01-01

**Authors:** Corrina Moucheraud, John Mboya, Doris Njomo, Ginger Golub, Martina Gant, May Sudhinaraset

**Affiliations:** a Fielding School of Public Health, University of California Los Angeles, Los Angeles, California, USA; b UCLA Center for Health Policy Research, University of California Los Angeles, Los Angeles, California, USA; c Innovations for Poverty Action, Kenya; d Kenya Medical Research Institute, Kenya; e Child.org, Kenya

**Keywords:** Child health, COVID-19, health services, maternal health, trust

## Abstract

We explore how the COVID-19 pandemic was associated with avoidance of, and challenges with, antenatal, childbirth and postpartum care among women in Kiambu and Nairobi counties, Kenya; and whether this was associated with a report of declined trust in the health system due to the pandemic. Women who delivered between March and November 2020 were invited to participate in a phone survey about their care experiences (n = 1122 respondents). We explored associations between reduced trust and care avoidance, delays and challenges with healthcare seeking, using logistic regression models adjusted for women’s characteristics. Approximately half of respondents said their trust in the health care system had declined due to COVID-19 (52.7%, n = 591). Declined trust was associated with higher likelihood of reporting barriers accessing antenatal care (aOR 1.59 [95% CI 1.24, 2.05]), avoiding care for oneself (aOR 2.26 [95% CI 1.59, 3.22]) and for one’s infant (aOR 1.77 [95% CI 1.11, 2.83]), and of feeling unsafe accessing care (aOR 1.52 [95% CI 1.19, 1.93]). Since March 2020, emergency services, routine care and immunizations were avoided most often. Primary reported reasons for avoiding care and challenges accessing care were financial barriers and problems accessing the facility. Declined trust in the health care system due to COVID-19 may have affected health care-seeking for women and their children in Kenya, which could have important implications for their health and well-being. Programs and policies should consider targeted special “catch-up” strategies that include trust-building messages and actions for women who deliver during emergencies like the COVID-19 pandemic.

## Introduction

Use of health services has declined worldwide during the COVID-19 pandemic.^1–8^ Analyses from Africa have found heterogeneous impacts across countries and outcomes: childhood immunizations declined in many countries, although extent, timing and duration of disruptions varied^9^; and there have been mixed results about changes in use of antenatal and childbirth services^10–12^—indicating that more in-depth and context-specific studies are needed.^13^

Studies from Kenya (the setting for this study) have identified a decreased volume of outpatient and inpatient visits compared to pre-pandemic levels,^14^ and declines in perinatal service use between March and December 2020 compared to expected service volume, with particular disruption in rural areas.^15^ A time trend analysis of Kenyan service utilization data also found that outpatient visits and childhood immunizations saw among the largest declines versus pre-pandemic levels, due both to the pandemic and the associated health workers strike—although use of antenatal care and facility-based delivery both declined during the strike but rebounded after.^16^

Barriers to the timely use of health services may be especially relevant for maternal and neonatal outcomes, as delayed care-seeking can be associated with increased risk of morbidity and mortality for these groups.^17–21^ During the 2014 West African Ebola outbreak, use of maternal and child health care services plummeted and likely resulted in substantial loss of life.^22–24^ The impact of the COVID-19 pandemic on maternal and neonatal health outcomes is not yet known: although some studies have suggested that these have worsened due to the pandemic,^25–27^ further research is needed to fully understand this relationship as many studies have relied on service-level data so do not capture outcomes that occur in the community,^28^ such as related to home births, for example, or avoided and averted care—both of which increased during the COVID-19 pandemic as described above.

There are many reasons why care-seeking may have changed during the COVID-19 pandemic, including: fears of contracting the virus at health facilities (as witnessed during previous Ebola outbreaks in Western Africa^29,30^); stockouts of key health goods due to global supply chain challenges, and consequently, impacts on service provision, including immunization^31^ and family planning^32^; and increased economic instability that may cause individuals to defer needed health care.^33,34^ Another factor may be decreased trust.^35–38^ Trust is a key ingredient for the provision of, and use of, high-quality and effective health care.^39–44^ Data from high-income countries has indicated that trust in institutions is associated with COVID-19 vaccine attitudes and uptake^45–48^; and during previous Ebola outbreaks in Africa, people with low institutional trust had lower health care utilization^49^—so trust is thus likely an important determinant of care-seeking, but its role during the COVIC-19 pandemic in Africa has not yet been well-characterized. We conceptualize trust as something that may directly affect care-seeking decisions and experiences, as well as something that may be impacted by—and may affect the impact of—other factors like access barriers. Previous studies have similarly identified trust in the health system as an important determinant of perinatal care utilization in Kenya.^50–52^

The objective of this manuscript is to examine perinatal care experiences–avoidance of care, and barriers seeking care–for women in two counties of Kenya who delivered between March and November 2020; and factors associated with these experiences including changing trust in the health system during COVID-19. (In March 2020, Kenya instated national movement restrictions and risk mitigation measures.)

## Methods

### Study Setting

By the end of 2020 (the time of this survey), Kenya had experienced approximately 96,000 cases of COVID-19 and 1670 deaths.^53^ There were two pandemic “surges” in Kenya during 2020, one during the months of July and August (peak of new infections at end of July), and another that began in October and subsided at the end of the year (peak of new infections in mid-November).^53^

Throughout 2020, Kenya introduced numerous restrictions on movement including curfews, intra- and inter-national transport limitations. From mid- to late-2020, there were frequent health worker strikes in Kenya due to frustrations about workload, burnout and inadequate protection against the virus.^54,55^ Kenya adjusted its maternal health care guidelines early during the COVID-19 pandemic—for example, suggesting that some antenatal visits could occur via phone rather than in-person (although still recommended eight visits)—there were no substantive changes to postpartum care guidance, and the only noteworthy change to immunization services was the cancelation of outreach campaigns (but routine immunization at health facilities was maintained as an essential service).^56^

### Sample Selection

This analysis uses data collected from a parent survey (detailed methods information available.^57^) In brief, six facilities were selected in Nairobi and Kiambu counties (three public hospitals, two private hospitals and one health center), and women aged 15–49 years who resided in the catchment areas of these facilities who had delivered a baby since March 2020 were identified by trained community health volunteers, who are assigned to deliver home-based essential maternal and neonatal care in defined geographic areas, so are familiar with the pregnancy status of women in their assigned areas. To be eligible, women needed access to a functional phone, to allow for mobile phone surveying. All women were surveyed between September and December 2020. In total, 2011 women were approached, of whom 233 were ineligible, contact could not be made with 618 (wrong phone number, or no answer), 11 women refused, and 14 began the survey but did not complete it—for a total survey sample size of 1135 women.

Previous analyses from the parent study (see below) found that 99% of women in this area attended antenatal care before COVID-19, and nearly two-thirds received four or more visits.^58^ This figure corresponds to the 2014 Demographic and Health Survey (DHS)—i.e., approximately 70% of women with recent births in Nairobi, and 67% in Kiambu county, received four or more antenatal visits—and the DHS also found that over 94% of women who recently delivered in Kiambu county, and over 90% of women who recently delivered in Nairobi county, delivered at a health facility.^59^

### Data Collection

Women were contacted by phone to assess eligibility, consent in the study if interested and eligible, and then participate in the survey. Those unavailable to complete the survey at the time of phone contact scheduled a follow-up appointment. Women not able to be contacted received up to nine phone call attempts on varying days of the week and times of day before being classified as unreached or a refusal. Surveys were conducted by eight experienced, female enumerators and one female supervisor, all of whom participated in an intensive, three-day virtual training, plus one day of pre-testing with 30 women. Verbal consent was obtained and audio-recorded prior to beginning the survey. Women who consented in the study received approximately 1 USD worth of mobile credit to appreciate their participation. The calls for women who completed the survey lasted (median) 32 minutes.

### Study Measures

The survey included questions about sociodemographic and health status, health care use and avoidance, and COVID-19 behaviors and attitudes.

All women were asked “In general, has your trust in the healthcare system improved, stayed the same, or declined due to COVID-19?” The main independent variable was dichotomous: did trust decline (yes, or no which included trust improved and trust stayed the same).

Outcome measures about care avoidance and challenge during the perinatal period are shown in Table 1. Some asked specifically about the COVID-19 pandemic while others did not.

### Data Analysis

Women whose baby had died between birth and time of the survey were excluded from the analysis (n = 13). We evaluated the characteristics of who avoided care since March 2020, and used adjusted logistic regression models to assess whether women with declined trust had different odds of each outcome variable. We also describe what care was reportedly avoided and by whom, and why care was avoided.

Covariates were selected as those potentially associated with care avoidance and its hypothesized relationship with trust: woman’s age, marital status (married or partnered, versus single, widowed or divorced), parity (first birth yes/no), educational attainment (completed secondary/attended college or university, versus some secondary or below), employment status (employed yes/no), self-reported health (excellent, very good or good, versus fair, poor or very poor), and month of childbirth. Models about postpartum care also included variables to capture previous care experiences during pregnancy and childbirth as these may influence trust^60^ and future care behaviors^61^: number of antenatal care visits (continuous); and a score representing person-centered maternity care, using a validated 30-item scale that measures women’s dignity and respect, communication and autonomy, and supportive care during maternity care^57,61–63^ (continuous). Postpartum care models also included presence of birth complications (yes/no), and infant postpartum care models included whether the birth was full-term (completed 37 weeks’ gestation or more, yes/no), as these may impact the need to subsequently seek care. All analyses were conducted using Stata v17.0.

### Ethical Review

Ethical clearance was received from the Kenya Medical Research Institute (KEMRI), Scientific and Ethics Review Unit (NON-KEMRI 702) and from the University of California Institutional Review Board (IRB #20–001421). A research permit was obtained from the government of Kenya through the National Commission for Science, Technology & Innovation (NACOSTI). Verbal consent was obtained from all the study participants.

## Results

A total of 1122 participants contributed data to this analysis. Their characteristics are shown in Table 2; women were 27.4 years on average, most were married or partnered (n = 760, 67.7%) and only 27.6% (n = 310) were nulliparous. Just under half of respondents had completed secondary school or attended college/university (n = 514, 45.8%). Approximately three-quarters of women were not working (n = 855, 77.7%), and most felt they were in excellent, very good or good health (n = 698, 62.2%). The average infant had been born 141 days prior (median: 141 days, 25th percentile 89 days, 75th percentile 199 days).

Slightly over half of respondents said that their trust in the health care system had declined due to COVID-19 (52.7%, n = 591). Approximately one-quarter of women said their trust had improved (26.7%, n = 299) and one-fifth said it had stayed the same (20.7%, n = 232). Reports of declined trust, versus staying the same or improving, were significantly more common (55.8%, n = 339) among those with less than secondary education compared to those with higher education (49.0%, n = 252) (Appendix Table A1) but there were no other significant differences by respondent characteristic.

### Care Avoidance and Reported Access Barriers

**Figure 1.**
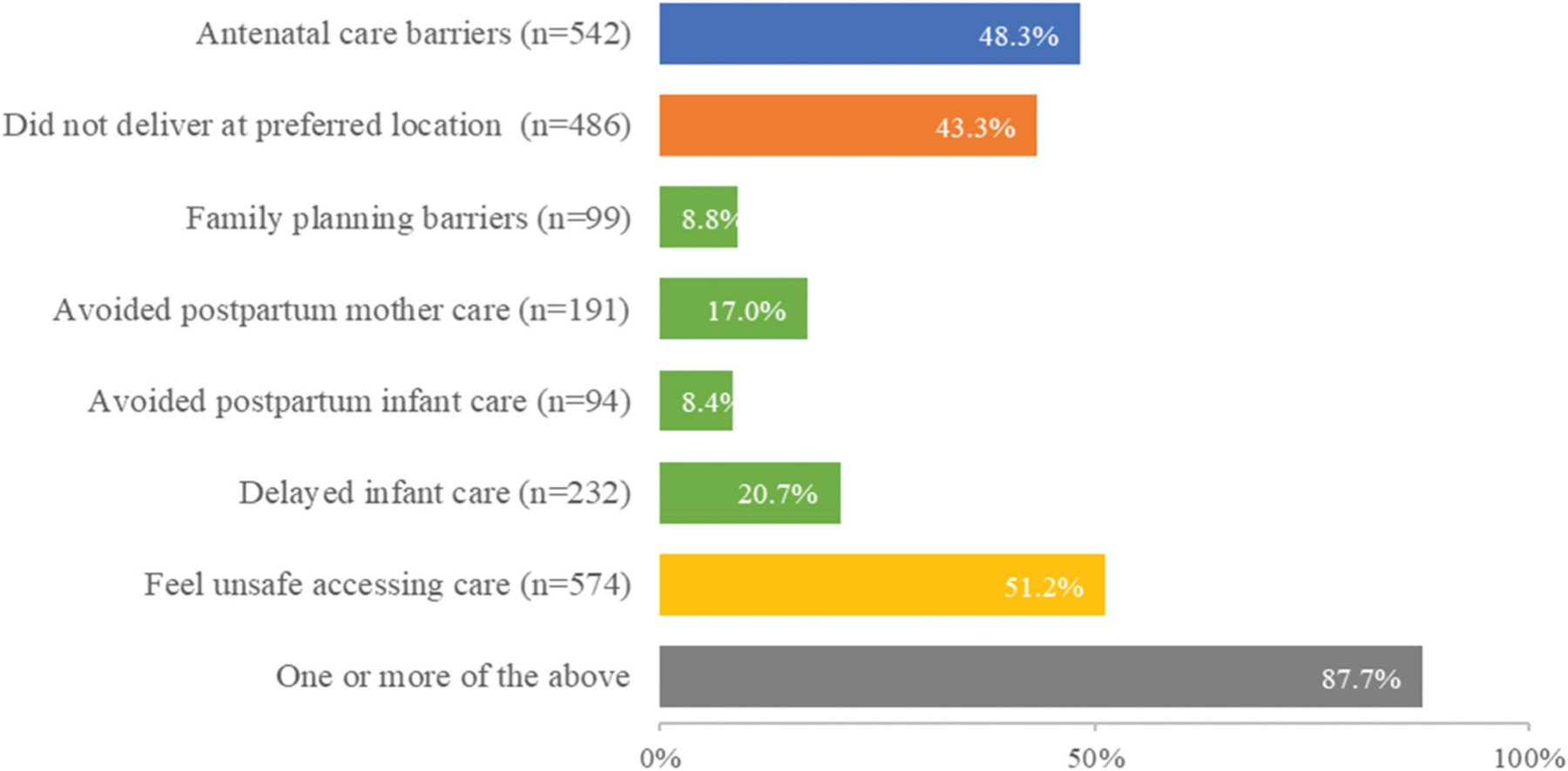
Percentage of women who reported care avoidance and barriers (n = 1122).

The majority of women reported at least one type of care avoidance or barrier: only 138 women (12.3% of the sample) said they faced no such problem (Figure 1). The most common issues reported were: feeling unsafe accessing care (51.2% of women, n = 574), barriers accessing antenatal care (48.3% of women, n = 542), and not delivering in one’s preferred location (43.3% of women, n = 486). (In this sample, 95% of women delivered at a health facility: only 48 women delivered at their or someone else’s home, and 12 delivered while en route to the hospital. Nearly all [95%] of the women who delivered at home or en route said it was not their preferred location, as did 42% of those who delivered at a health facility.) Overall, 8.4% reported avoiding postpartum infant care, 17.0% reported avoiding care for themselves.

Among those who reported needing but avoiding care for themselves or their infant since March 2020 (n = 191 and n = 94 respectively), women were asked which services they had avoided (Table 3). Approximately half of women who reported avoiding care for themselves said they had avoided postpartum emergency care; whereas the most common types of care avoided for infants were immunizations and routine care/checkup visits (each was reported by just under half of women who said they had avoided care for their infant since March 2020).

### Reasons for Care Avoidance

Women were asked why they had avoided services (Table 4). Sixty-one percent of women reporting an antenatal care access barrier, 46% of women who said they delayed immunizations or routine care for their infant, approximately 30% of women who reported that they did not deliver at their preferred location, and approximately 30% of women who avoided infant care said that facility access was a challenge–e.g., facilities being too busy, facilities being closed, or health workers being unavailable. The most common challenge with family planning access was a stockout of supplies/commodities (reported by approximately half of women who had a problem with this service). Financial barriers—i.e., not being able to afford care—was the most frequently-reported challenge for women who avoided postpartum care for themselves and their infants (reported by 41% and 29% of these women, respectively) and was also reported by approximately 20% of women who faced antenatal care barriers and who faced family planning care barriers.

Across all types of services, the most common reason for care avoidance or care barrier was related to facility access (reported by approximately half of women reporting any avoidance or of problem with care). Approximately one-quarter of women who avoided care or had an access challenge said this had been due to a financial challenge, 17.5% attributed this to fear of COVID-19 contagion, and 13.7% said it was because of a COVID-related restriction like curfew or need to purchase personal protective equipment. Nearly every reason was more commonly reported by women who said their trust in the health system had declined due to COVID-19 (compared to women who said their trust had remained the same or improved), but these differences were mostly small and not statistically significant, except financial barriers and COVID-related restrictions which were reported significantly more often by women who said their trust had declined (Appendix Table A2).

### Correlates of Care Avoidance/Barriers

Married women and women with below-secondary education were less likely to report having faced problems accessing antenatal care and feeling unsafe accessing care, and women with better self-reported health less commonly faced antenatal or family planning barriers, as well as avoidance of postpartum care for themselves (Appendix Table A3). No other demographic characteristics were associated with these outcomes.

Declining trust was strongly associated with care avoidance (Table 5). In models including all covariates, those who reported that their trust in the health system had declined due to COVID-19 had 58% higher odds of reporting antenatal care barriers, 124% higher odds of reporting avoidance of postpartum mother care, 73% higher odds of reporting avoidance of postpartum infant care, and 50% higher odds of reporting feeling unsafe accessing medical care. Appendix Table A4 presents all coefficients for all variables (explanatory and covariates) in the model fit for each outcome.

## Discussion

Nearly all respondents in this study of women in Nairobi and Kiambu counties (Kenya) who delivered during 2020 reported a challenge with care-seeking or reported care avoidance during the perinatal period. In addition, approximately half of women in the study reported that their trust in the health system had declined due to COVID-19, and this was associated with avoidance of care, and with reporting of barriers and challenges in accessing care. Declining trust may be a cause of care avoidance or care challenges, or may be a consequence of it—but in either case, efforts to improve trust in healthcare systems are needed particularly in response to the COVID-19 pandemic and other pandemics.

Trust is an ingredient of health services decision-making that merits urgent attention: in a global survey, only a quarter of respondents indicated that they had a lot of trust in their government, and trust was associated with trust in health and medical advice.^64^ Despite its importance and a growing literature from high-income countries during the COVID-19 pandemic,^45–48,65–67^ the relationship between trust and health behaviors (and, ultimately, outcomes) remains relatively under-investigated in the African context. This paper contributes to filling that gap.

Women commonly reported challenges accessing antenatal care; a previous survey among pregnant women in Kenya found that 21% of them planned to avoid antenatal care visits^68^ but our finding may be higher because it reflects actual—in addition to anticipated—care avoidance. In addition, over 43% of women in this survey did not deliver at their preferred location. Nearly all women in the study sample delivered at a health facility (95% of those surveyed)—which matches an overall trend in Kenya of increased facility delivery^69,70^; it is therefore possible that women wanted a facility-based delivery but the exact location did not match their preference. Although many women said they did not deliver at their preferred location due to clinical reasons (emergency delivery, or referral from doctor), many women cited facility-level factors of closures, being at capacity, and health worker strike.^71^

Avoidance of care during the postpartum period was less common—but among those who reported this, approximately half of women said they had avoided emergency care, and just under half said they had avoided routine infant care/checkups and immunizations. These findings correspond with other research from across Africa: perinatal healthcare access has been disrupted during the COVID-19 pandemic.^10,11,13,15,58,72–78^

The most common challenges and reasons for care avoidance were facility access barriers and financial constraints. Facility access took the form of concerns about facility closures, provider strikes and being turned away from care. Financial constraints included inability to afford care and pay for transportation to the facility. Access to health services in Kenya was challenging for women and infants even prior to 2020,^79–81^ but many of these factors have been exacerbated by the pandemic. This mix of patient- and system-side factors has parallels in studies on HIV services in Kenya during the COVID-19 pandemic, which were affected by financial constraints (exacerbated by unemployment due to the pandemic), health workers diverted to other services, and curfews leading to limited facility hours of operation.^82,83^ In a multi-national study, respondents in Africa were much more likely to attribute foregone medical care during COVID-19 to financial concerns (rather than COVID-19, access or other reasons) than respondents from other regions.^84^ There were also stockouts of key medical commodities, which impacted care-seeking—both in our study and in previous studies from Kenya.^85^ The pandemic has also had catastrophic effects on women’s livelihoods, including those in precarious and informal sectors,^86^ which is likely to be particularly acute for pregnant and postpartum women who may not have paid parental leave or employment security following childbirth. Postpartum women contend with hospital fees related to the pandemic—such as being required to pay a fee to cover the cost of PPE during intrapartum and postpartum visits, which exacerbates employment-related impacts of the pandemic and household experiences of food insecurity, and is likely to influence healthcare seeking.^87^ A qualitative study with Kenyan people living in slum communities similarly found that financial barriers due to COVID-19—including the cost of acquiring PPE and lost wages that increase economic precarity—deterred care-seeking.^85^ Health worker strikes at public-sector facilities in Kenya may also have had a particularly severe impact on lower-income women. Future studies might seek to assess how financial challenges and burden interact with access to health services to affect care avoidance and experiences during emergencies.

This study has limitations that should be noted. First, the measure of decline in trust is limited as we only asked one question. Future studies should include validated multi-dimensional measures of trust to comprehensively examine how trust in healthcare system is associated with care avoidance. Second, the main sample of women had infants ranging from 0 to 36 weeks old. Some women were therefore reporting on antenatal or childbirth care that had only recently occurred, while others were recalling over a longer period. Additionally, women who delivered earlier had a longer “exposure” period, i.e., could report on more opportunities for care avoidance during the postpartum period. Third, we could not disentangle the period and cohort effects. It is possible that women with younger, or older, infants make different decisions and would be affected by the COVID-19 pandemic in different ways; and it is possible that negative pandemic-related experiences accumulate over time. Similarly, the effects of the COVID-19 pandemic may be felt in waves, as emerging variants cause burden to fluctuate dramatically, and as policies such as movement restrictions and masking requirements can change over time.^33^ Lastly, these results should be generalized with caution as the women surveyed may differ from other populations in key ways including mobile phone ownership and universal awareness of the COVID-19 pandemic.

The study has a number of policy and practice implications. In line with Kenya’s community health strategy 2020–2025,^88^ local governments may work to strengthen community health and volunteer (CHV) networks, and leverage them particularly during emergencies.^89^ CHVs make home visits, deliver health information and education, and treat common illnesses.^90^ CHVs are supervised monthly by government employees, known as Community Health Extension Workers, who serve to link households to health facilities.^90^ Community health volunteers are potentially an important conduit between health facilities and communities, but were underutilized in Kenya at the time of this study. Investments in community-based healthcare has the potential to rebuild trust by engaging women and their newborns who may have missed or delayed healthcare.^91–93^ However, studies from Africa have found that trust in CHVs is variable, and that factors like health worker support (or, conversely, rejection) of CHV credibility significantly influenced women’s trust in CHVs.^94^ Further research on how to leverage community health workers, and special considerations for this during emergencies, is urgently needed. Future studies should examine trust in CHVs, and its correlates, using validated measures.^95^ Additionally, healthcare facilities have an important role to play in rebuilding trust and providing updated information on COVID-19 to improve the health of mothers and newborns. For example, providers and healthcare staff should be trained on person-centered maternity care to provide care that is respectful of and responsive to women’s and their families’ preferences, needs, and values—including attention to how these may change during an emergency (like the COVID-19 pandemic). This includes training providers on calling women by their names, introducing themselves, and ensuring women have autonomy during their care.^96^ Additionally, interventions that center supportive care throughout the process of labor and delivery may improve respectful care.^97^ Particularly during emergency situations, person-centered care needs to extend health facility walls to ensure continuity of care from, and to, communities. The inclusion of community health volunteers, or using technology to link women to the health system, should be explored as approaches that may meet women’s needs and preferences. Global surveys have found that health works who provide perinatal care are experiencing negative psychological impacts, including due to increased workload and stress^98^; and consequently are finding it harder to provide respectful maternity care during the COVID-19 pandemic than before.^99^ This suggests the importance of also supporting health workers’ needs during emergencies so they can offer the highest possible quality of care to women.

## Conclusions

The perinatal period is a critical and vulnerable time for women and their children, and it is a period when many women engage consistently and frequently with the health system. During pandemics and other emergencies, dedicated efforts are needed to ensure that pregnant and postpartum women remain engaged in care. Building and maintaining trust in the health system is essential for reaching this key group, and should be accompanied by other interventions as suggested by this study—such as clear communication about facility operating hours when these are changed, strong linkages and referrals across facilities when women are turned away, greater use of community-based care to reduce congestion at health facilities and to lessen the economic burden of transport for care-seeking, and ensuring strong supply chains of commodities during emergencies.

## Figures and Tables

**Table 1.** Outcome measures based on survey questions.

Survey question	Operationalization for this analysis
How did COVID-19 affect your ability to access antenatal care or attend antenatal care appointments?	Antenatal care barriers: *0: No impacts* *1: Any reported impact(s)*
For your most recent delivery, where did you give birth? Was this where you preferred or intended to give birth?	Did not deliver at preferred location: *0: Was where I preferred/intended* *1: Was not where I preferred/intended*
Have you experienced any issues when trying to receive or obtain a family planning method since COVID (mid-March)?	Family planning barriers: *0: No* *1: Yes*
Since your delivery, have you needed care but avoided using or were otherwise unable to use health services or visit health care providers?^†^	Avoided postpartum mother care: *0: No* *1: Yes*
Since your delivery, have/had you avoided or delayed taking your baby to visit health care providers or use health care services? ^†^	Avoided postpartum infant care: *0: No* *1: Yes*
Did you ever have to miss or present late to any immunizations or recommended clinic visit for any of the following reasons? ^†^	Delayed infant care: *0: Never missed or presented late* *1: Any reported reason(s)*
To what extent do you agree or disagree with: I feel unsafe going to a health center because of the COVID-19 outbreak. ^†^	Feel unsafe accessing care: *0: Strongly disagree or disagree* *1: Strongly agree or agree*

† also included in sensitivity analysis; rather than “Since your delivery … “ these questions asked “Since mid-March … “

**Table 2.** Characteristics of the sample (n = 1122).

	n (%)
Age, average (median)	27.4 (27)
Married or partnered: Yes	760 (67.7%)
Not married/partnered (single, widowed, divorced)	362 (32.3%)
First birth: Yes	310 (27.6%)
> 1 parity	812 (72.4%)
Educational attainment: Some secondary or below	608 (54.2%)
Completed secondary, attended college or university	514 (45.8%)
Employed (full or part time, formal or informal sector): No	855 (77.7%)
Yes	246 (22.3%)
Self-reported health: Fair, Poor, or Very poor	424 (37.8%)
Excellent, Very good, or Good	698 (62.2%)
Age of child at time of survey, average (median) weeks	20.2 (20.1)
Trust in the health system: Declined due to COVID-19	591 (52.7%)
Stayed the same	232 (20.7%)
Improved	299 (26.7%)

**Table 3.** What services were avoided since March 2020?

	Avoided postpartum mother care (n = 191)	Avoided postpartum infant care (n = 94)
Emergency care	95 (49.7%)	29 (30.9%)
Routine care/checkup	32 (16.8%)	42 (44.7%)
Immunizations	n/a	41 (43.6%)
Acute care	19 (10.0%)	4 (2.3%)
Family planning	16 (8.4%)	n/a
COVID-19 test	9 (4.7%)	n/a
Dental care	4 (2.1%)	n/a
Pharmacy	3 (1.6%)	n/a
Postnatal care	4 (4.2%)	n/a

**Table 4.** Reasons for avoiding each type of care (n = 1122) (percentage is among those women reporting that type of challenge/avoidance).

	Antenatal care barriers (n = 542)	Did not deliver at preferred location (n = 486)	Family planning barriers (n = 99)	Avoided postpartum mother care (n = 191)	Avoided postpartum infant care (n = 94)	Delayed infant care (n = 232)
COVID-19 contagion fears	122 (22.5%)	14 (2.9%)		48 (25.1%)	12 (12.8%)	8 (3.4%)
Financial barriers	98 (18.1%)	71 (14.6%)	21 (21.2%)	79 (41.4%)	27 (28.7%)	19 (8.2%)
Facility access barriers	331 (61.1%)	143 (29.4%)	22 (22.2%)	38 (19.9%)	31 (33.0%)	107 (46.1%)
COVID-related restrictions (PPE, curfews, etc.)	68 (12.%)	63 (13.0%)		4 (2.1%)	7 (7.4%)	
Lack of transport	2 (0.4%)	21 (4.3%)		4 (2.1%)		8 (3.4%)
Felt ill	1 (0.2%)			5 (2.6%)	2 (2.1%)	19 (8.2%)
COVID-related stigma (fear of testing)				5 (2.6%)		
Referred elsewhere by health care worker		111 (22.8%)				
Emergency birth, went to nearest facility		82 (16.9%)				
Stockouts/shortages			50 (50.5%)			28 (12.1%)
No time to go				6 (3.1%)	14 (14.9%)	21 (9.1%)
Other	21 (3.9%)	17 (3.5%)	14 (14.1%)	17 (8.9%)	4 (4.3%)	36 (15.5%)

Women could cite more than 1 reason for each care type.

**Table 5.** Adjusted odds of reporting care barriers or care avoidance for those whose trust in the health system declined due to COVID-19, compared to those whose trust remained the same or improved (n = 1122).

	aOR (95% CI)
Antenatal care barriers	1.58*** (1.23, 2.02)
Did not deliver at preferred location	1.20 (0.94, 1.53)
Family planning barriers	1.39 (0.90, 2.16)
Avoided postpartum mother care	2.24*** (1.57, 3.19)
Avoided postpartum infant care	1.73* (1.09, 2.75)
Delayed infant care	1.35 (0.98, 1.84)
Feel unsafe accessing care	1.50** (1.18, 1.92)

One row represents one model.

Includes covariates: age (continuous), marital status (single/widowed/divorced, or married/partnered), parity (> 1, or first birth), educational attainment (completed secondary/attended college or university, or some secondary or below), employment status (employed full or part time, formal or informal sector, Yes or no), self-reported health (fair/poor/very poor, or Excellent/very good/good), and delivery month. Avoided postpartum care (mother and infant) and delayed infant care models also include number of ANC visits (less than 4, 4–7, 8+); avoidance of postpartum maternal care includes person-centered maternity care score (continuous) and presence of delivery complications (yes or no); avoidance of postpartum infant care and delayed infant care include full-term delivery (yes [weeks 38+], or no [< 38 weeks]).

* *p* < .05

** *p* < .01

*** *p* < .001.

## Data Availability

The data underlying this article will be shared on reasonable request to the corresponding author.

## Appendix

**Table A1.** Whose trust has declined.

	Trust improved or stayed same	Trust declined	*p*-val on chi2
Age: <25	186 (47.3%)	207 (52.7%)	.99
Age 25+	345 (47.3%)	384 (52.7%)	
Married or cohabitating/partnered: Yes	359 (47.2%)	401 (52.8%)	.93
Not married (single, widowed, divorced)	172 (47.5%)	190 (52.5%)	
First birth: Yes	156 (50.3%)	154 (49.7%)	.21
> 1 parity	375 (46.2%)	437 (53.8%)	
Educational attainment: Some secondary or below	269 (44.2%)	339 (55.8%)	.02
Completed secondary, attended college or university	262 (51.0%)	252 (49.0%)	
Employed (full or part time, formal or informal sector): No	394 (46.1%)	461 (53.9%)	.19
Yes	125 (50.8%)	121 (49.2%)	
Self-reported health: Fair, Poor, or Very poor	185 (43.6%)	239 (56.4%)	.053
Excellent, Very good, or Good	346 (49.6%)	352 (50.4%)	

**Table A2.** Among women reporting any negative care experience (*n* = 984), those reporting each reason for challenge/avoidance, and association with reported trust.

	Full sample	Among those who said that their trust same/improved	Among those who said that their trust declined	*p*-val (chi2)
Facility access barriers	499 (50.7%)	217 (48.4%)	282 (52.6%)	.19
Financial barriers	229 (23.3%)	88 (19.6%)	141 (26.4%)	.01
COVID-19 contagion fears	172 (17.5%)	75 (16.7%)	97 (18.2%)	.56
COVID-related restrictions (PPE, curfews, etc.)	135 (13.7%)	45 (10.0%)	90 (17.0%)	.002
Stockouts/shortages	77 (7.8%)	33 (7.4%)	44 (8.2%)	.62
Lack of transport	34 (3.5%)	17 (3.8%)	17 (3.2%)	.59
No time to go	29 (3.0%)	12 (2.7%)	17 (3.2%)	.65
Felt ill	23 (2.3%)	7 (1.6%)	16 (3.0%)	.14

Women could cite more than one problem.

*p*-value based on chi-square test comparing those who said their trust in the health system declined due to COVID-19 compared to those who said their trust improved or stayed the same.

**Table A3.** Who avoided and faced barriers, by characteristic, unadjusted odds ratios (standard errors).

	Antenatal care barriers	Did not deliver at preferred location	Family planning barriers	Avoided postpartum mother care	Avoided postpartum infant care	Delayed infant care	Feel unsafe accessing care
Age 25+ (ref)							
Age <25	1.12 (0.14)	0.81 (0.10)	1.23 (0.27)	1.00 (0.17)	0.91 (0.21)	0.88 (0.14)	1.08 (0.14)
Not married (single, widowed, divorced) (ref)							
Married or cohabitating/partnered	0.72* (0.09)	0.92 (0.12)	1.30 (0.31)	0.88 (0.15)	1.07 (0.25)	0.76 (0.12)	0.77* (0.10)
Parity > 1 (ref)							
First birth	1.04 (0.14)	1.30* (0.17)	0.78 (0.19)	1.07 (0.19)	1.00 (0.24)	1.05 (0.17)	1.25 (0.17)
Educational attainment: Completed secondary, attended college or university (ref)							
Some secondary or below	0.62*** (0.08)	0.90 (0.11)	1.22 (0.26)	0.96 (0.15)	0.96 (0.21)	0.92 (0.14)	0.78* (0.09)
Employed (full or part time, formal or informal sector): No (ref)							
Yes	0.93 (0.13)	0.96 (0.14)	1.21 (0.30)	0.79 (0.16)	1.29 (0.32)	1.31 (0.22)	0.98 (0.14)
Self-reported health: Fair, Poor, or Very poor (ref)							
Excellent, Very good, or Good	0.53*** (0.07)	0.92 (0.11)	0.41*** (0.09)	0.45*** (0.07)	0.70 (0.15)	0.79 (0.12)	0.88 (0.11)

* *p* < .05

** *p* < .01

*** *p* < .001.

**Table A4.** Full model details, adjusted odds ratio (95% CI).

	Antenatal care barriers	Did not deliver at preferred location	Family planning barriers	Avoided postpartum mother care	Avoided postpartum infant care	Delayed infant care	Feel unsafe accessing care
Trust declined (vs., trust stayed same or increased)	1.58*** (1.23, 2.02)	1.20 (0.94, 1.53)	1.39 (0.90, 2.16)	2.24*** (1.57, 3.19)	1.73* (1.10, 2.75)	1.34 (0.98, 1.84)	1.50** (1.18, 1.92)
Age (continuous)	0.99 (0.96, 1.01)	1.04** (1.01, 1.06)	0.97 (0.93, 1.02)	1.00 (0.96, 1.03)	1.03 (0.98, 1.07)	1.01 (0.98, 1.04)	1.00 (0.98, 1.03)
Married/cohabitating/partnered (vs., single/widowed/divorced)	0.67** (0.51, 0.88)	0.97 (0.74, 1.27)	1.48 (0.86, 2.48)	0.98 (0.67, 1.43)	1.18 (0.72, 1.96)	0.81 (0.57, 1.13)	0.74* (0.57, 0.98)
First birth (vs., parity > 1)	0.81 (0.58, 1.14)	1.58** (1.13, 2.21)	0.80 (0.45, 1.40)	1.00 (0.64, 1.58)	1.15 (0.62, 2.12)	1.02 (0.67, 1.57)	1.17 (0.84, 1.64)
Some secondary education or below (vs., completed secondary/attended college or university)	0.53*** (0.41, 0.69)	0.97 (0.75, 1.25)	1.18 (0.75, 1.87)	0.89 (0.62, 1.25)	1.02 (0.62, 1.68)	0.87 (0.63, 1.22)	0.79 (0.61, 1.02)
Employed (full or part time, formal or informal sector) (vs., not employed)	0.92 (0.67, 1.25)	0.94 (0.69, 1.29)	1.28 (0.73, 2.23)	0.73 (0.47, 1.15)	1.02 (0.60, 1.73)	1.16 (0.79, 1.69)	0.94 (0.69, 1.28)
Self-reported health excellent/very good/good (vs., fair/poor/very poor)	0.53*** (0.41, 0.68)	0.90 (0.70, 1.17)	0.42*** (0.27, 0.65)	0.46*** (0.33, 0.65)	0.75 (0.47, 1.19)	0.88 (0.64, 1.22)	0.93 (0.72, 1.20)
4–7 ANC visits (vs., less than 4)	*Not included*	*Not included*	*Not included*	0.98 (0.68, 1.41)	0.78 (0.48, 1.26)	0.82 (0.58, 1.14)	*Not included*
8+ ANC visits (vs., less than 4)	*Not included*	*Not included*	*Not included*	1.48 (0.66, 3.28)	0.77 (0.28, 2.12)	0.95 (0.44, 2.06)	*Not included*
PCMC score (continuous)	*Not included*	*Not included*	*Not included*	0.99 (0.97, 1.01)	1.02 (0.99, 1.05)	0.98 (0.96, 1.00)	*Not included*
Full-term delivery (vs., < 38 weeks)	*Not included*	*Not included*	*Not included*	*Not included*	0.51** (0.31, 0.82)	0.83 (0.57, 1.19)	*Not included*
